# Current accounts of antimicrobial resistance: stabilisation, individualisation and antibiotics as infrastructure

**DOI:** 10.1057/s41599-019-0263-4

**Published:** 2019-05-21

**Authors:** Clare I. R. Chandler

**Affiliations:** Department of Global Health and Development, London School of Hygiene & Tropical Medicine, 15-17 Tavistock Place, London, WC1H 9SH UK

**Keywords:** Social anthropology, Science, technology and society

## Abstract

Antimicrobial resistance (AMR) is one of the latest issues to galvanise political and financial investment as an emerging global health threat. This paper explores the construction of AMR as a problem, following three lines of analysis. First, an examination of some of the ways in which AMR has become an object for action—through defining, counting and projecting it. Following Lakoff’s work on emerging infectious diseases, the paper illustrates that while an ‘actuarial’ approach to AMR may be challenging to stabilise due to definitional and logistical issues, it has been successfully stabilised through a ‘sentinel’ approach that emphasises the threat of AMR. Second, the paper draws out a contrast between the way AMR is formulated in terms of a problem of connectedness—a ‘One Health’ issue—and the frequent solutions to AMR being focused on individual behaviour. The paper suggests that AMR presents an opportunity to take seriously connections, scale and systems but that this effort is undermined by the prevailing tendency to reduce health issues to matters for individual responsibility. Third, the paper takes AMR as a moment of infrastructural inversion (Bowker and Star) when antimicrobials and the work they do are rendered more visible. This leads to the proposal of antibiotics as infrastructure—part of the woodwork that we take for granted, and entangled with our ways of doing life, in particular modern life. These explorations render visible the ways social, economic and political frames continue to define AMR and how it may be acted upon, which opens up possibilities for reconfiguring AMR research and action.

## Introduction

‘Due to antimicrobial resistance, many achievements of the twentieth century are being gravely challenged, in particular: the reduction in illness and death from infectious diseases achieved through social and economic development; access to health services and to quality, safe, efficacious and affordable medicines; hygiene, safe water and sanitation; disease prevention in community and health-care settings, including immunisation; nutrition and healthy food; improvements in human and veterinary medicine; and the introduction of new antimicrobial and other medicines’ (Item 4, Draft Political Declaration on AMR, United Nations General Assembly, 2016)

Reports of infections that are non-responsive to first-line antimicrobials have increased. Concern over resistance of the microbes causing these infections to the drugs used to treat them has expanded as awareness of the absence of replacement medicines has risen (Tripartite Collaboration on AMR, 2016). As the field of antimicrobial resistance (AMR) research, policy and action grows, what patterns of knowledge and ways of knowing are shaping conceptual possibilities and boundaries? If these systems of thought and knowledge are explicated, how might the boundaries of these epistemes be pushed?

Sensitive to the controversy around the science of climate change (Pearce et al., 2018), those engaged with AMR advocacy have been careful to develop a ‘political narrative’ (Global Health Security Agenda, 2016) that comprises consistent messaging and a coherent voice within a science-policy complex. The collaboration across the ‘tripartite’ of the World Health Organisation (WHO), Food and Agriculture Organisation (FAO) and the World Organisation for Animal Health (OIE) has been commended as providing a platform for successful intervention across UN member states (Inter Agency Coordination Group on AMR Consultation, 2018). An indicator of success of getting AMR onto the political agenda was the high-level discussion of AMR at the United Nations Global Assembly in 2016, resulting in a political declaration (United Nations General Assembly, 2016), and the appointment of an Inter-agency Coordination Group (IACG) on AMR which seeks to generate a common direction across sectors, industry and nations globally. This effort has been credited with enabling strong global consensus on the problem of AMR and has garnered political will to address the threat (Rochford et al., 2018). Industry leaders in pharmaceuticals and diagnostics have become partners in the fight against AMR (Wellcome Trust, 2017), articulated for example in a Davos declaration (AMR Industry Alliance, 2016). The successful generation of this collective commitment is said to hinge on the use of common language (Mendelson et al., 2017) combined with a shared approach and set of concerns (Inter Agency Coordination Group on AMR, 2017).

While AMR has been successfully established as a priority on the global stage, AMR champions repeatedly voice concern over the ‘implementation challenge’ of translating political commitment into action (Wells et al., 2017, UK Department of Health, 2017). Thus far, standardised frameworks and tools are promoted for country-level implementation, but ‘there is still much more that needs to be done’ (Inoue and Minghui, 2017). Discourse analysis can explicate to the way AMR has been stabilised and mobilised as an object, can bring to the fore consequences of the way the AMR problem has been framed, and can open up other ways to attend to the constellation of issues represented by AMR.

## Approach

In this paper I intend to bring to the fore aspects of the AMR agenda that are taken for granted and deemed self-evident as research, policy and action has been taking shape. The primary inspiration for the approach is a Foucauldian discourse analysis, through which the object of AMR is explored in how it is represented and how it is practiced—treating discourse ‘as practices that systematically form the objects of which they speak’ (Foucault, 1972, p. 49). AMR might usefully be considered such an object of discursive formation, contingent upon relations ‘between institutions, economic and social processes, behavioural patterns, systems of norms, techniques, types of classification, modes of characterisation’ (p. 45). To trace AMR in this way then develops analysis that understands it as an object that emerges in relation to particular rationalities, juxtaposed with other objects that define its difference.

Recognising the global nature of the AMR discourse, the numerous institutions, connections and imperatives can be considered to comprise a ‘global assemblage’, hanging together under the umbrella of AMR (Ong and Collier, 2005). In attending to this emergent ‘thing’ of AMR, I follow the move to consider diseases as socio-materially co-constructed entities, whose form is enacted in multiple ways, inspired by the work of Mol (2002). This sets the scene for my own understanding of what AMR is, which is then built upon by observing the discourse in ‘the AMR Community’ (an entity reified within the Community of scientists, policy and public/global health practitioners) through analytical lenses borrowed principally from Lakoff (2017), Rose (2001) and Bowker and Star (2000).

Through this analysis, I present three current accounts of antimicrobial resistance (AMR) as it has emerged in relation to particular priorities and formulations of science and policy. First, I focus on the challenges of stabilising AMR as an object through traditional definitions and counting, and explore the way that projection of AMR as a threat seems to have been more effective for stabilising this object. Second, I juxtapose two core features of the AMR risk discourse—the One Health paradigm, and the behavioural model of intervention—drawing attention to a paradox in AMR as a problem of connectedness to be solved by individualised action. Third, I develop an account of AMR that conceives of antibiotics as infrastructure, opening-up alternative paths for exploring this emergent field.

The analysis draws from a range of material that includes published scientific and policy literature, scientific conference presentations and interviews and discussions with scientists and policy makers. The analysis is informed by my own immersion in the field of AMR science and policy, as a researcher and as co-Director of the AMR Centre at the London School of Hygiene & Tropical Medicine.

Ethical approval for the fieldwork in this study was granted by the LSHTM Observational Ethics Committee, reference numbers 11598 and 15244.

## Stabilising antimicrobial resistance as a threat

‘There is a gesture often used to describe the antimicrobial resistance (AMR) problem—an inflection of the hand tracing a curve like the up-side of a mountain. At a meeting of scientists, a journalist asks, ‘How afraid should we be of AMR?’. This gesture provides the answer. At an AMR policy event, a civil servant is making the case for investment in AMR, he makes the gesture. I ask a medical doctor in a low-income country, ‘what is your experience of AMR where you practice?’. The same gesture. I see it repeating in settings of science, policy and practice around the globe. I can’t find the curve drawn out in the scientific literature, but we have a shared understanding that this is the shape of the curve. The gesture stands in place of data, communicating the need for response. It mirrors the ‘hockey stick curve’ deployed in the 2014–2015 Ebola response (Kelly, 2018), implicitly connecting the two crises. How has this shared understanding of the AMR threat been brought into being?’

*Fieldnotes* *London 15 and East Africa 12, 2018*

Lakoff (2015, 2017) proposes the concept of the ‘sentinel device’ to describe the way that public health threats have come to be responded to—requiring vigilant attention and speculative intervention for a surprise and potentially catastrophic event. He contrasts this with the more established ‘actuarial’ style of reasoning that justifies action through the statistical calculation of risk, projecting the past into the future. Actuarial devices work in a world where threats can become known through accumulation of epidemiological risk data and application of cost-effectiveness analyses to guide intervention. By contrast, sentinel devices work in a world where a dangerous future cannot be known through calculation and a precautionary mode is required to guide intervention. Observing the way topics in global health now often follow the speculative turn in finance, hedging on ‘potential’ rather than ‘likely’ events, affects the orientation of action including flows of both public and private finance. In each case, a distinct set of logics connects through particular modelling approaches that render financial, health and other (see also disaster and development discourses) paths for action more, and less, inevitable.

Here I will explore the way that the actuarial approach to AMR continues to be a strong part of AMR research and implementation agendas—to come to know where AMR is, how much there is and points for control—but that the definitional and technical challenges inherent to this complex category mean that this is challenging to stabilise as an object through this lens. By applying the logic of the sentinel, or speculative, device, it becomes possible to see how AMR has become stabilised on the global stage as a threat, and rendered amenable to particular forms of action—and investment.

### The actuarial approach: defining and counting AMR

Histories of AMR identify waves of political enthusiasm in the topic. The troughs between the waves have been attributed to complacency (Podolsky, 2018) but also to the complexity of the challenge of understanding and responding to this biologically inevitable and remarkably diverse issue (Wells et al., 2017). Attempts to stabilise AMR through actuarial accounts, in order to act upon it as an issue, have been challenged by difficulties in defining what AMR is, and in being able to count it.

Although AMR is a commonly used term, it is not easy to create a common definition. Broadly, it is used to describe a phenomenon of microbial organisms becoming less susceptible to the antimicrobial medicines used to eliminate or control them. This phenomenon is described in bacteria, fungi, parasites and viruses (see, e.g., Fisher et al., 2018; Dondorp et al., 2009; Keshavjee and Farmer, 2012; Laxminarayan et al., 2013; Baggaley et al., 2010). Amongst bacteria alone, the term AMR describes a wide range of species and strains which develop or acquire different characteristics that render medicines less effective. Mechanisms described as conferring antibiotic resistance vary widely, including enzyme production that inactivates the drug; modification of drug targets sites, preventing binding; reduction of permeability to the drug; production of alternative metabolic pathways that bypass the drug’s target pathway; and the use of various pumps to export antibiotics or prevent the drug from reaching a bacterial intracellular target (Kapoor et al., 2017). A given bacteria may have evolved a resistance mechanism or may have acquired it through transfer of genetic material from another bacteria of the same or even another species (Gillings, 2017). When an antibiotic is unable to act upon a bacteria because of that organism’s physical characteristics, this is also termed resistance, this time ‘intrinsic’ (Neu, 1992). The term AMR therefore accommodates a variety of microbial and genetic activity, even just within bacterial AMR. Similarly there are numerous registers for recognising AMR. Clinically, AMR may be recognised if a patient’s infection does not clear after an antibiotic expected to impact that infection is taken. Microbiologically, AMR may be recognised if bacteria that are grown from a sample and plated on agar in a laboratory are unaffected when an antibiotic is introduced. Molecularly, AMR may be recognised from the presence of a genetic sequence that has previously been typed as conferring drug resistance may be identified in a bacterial sample, or in a faecal, soil, water or other sample. And the ways we have come to understand resistance have evolved together with laboratory technology (Gradmann, 2013). When we talk of increasing resistance, then, we may be referring to an increase in clinical cases that are hard to treat, increase in identification of a particular bacteria with resistance to a particular drug, a bacteria resistant to a particular group of similar action drugs, a bacteria resistant to a range of drug types, or an increase in identification of a particular piece of genetic material. To complicate things further, carriage of any of these bacteria may or may not cause disease in the short or long term for the human carrier. And the existence of such bacteria in the metabiome—matter outside of humans—may also be ‘friendly’ or not. Therefore, while bacteria with a particular ability to resist particular antibiotics may be common in a given context, the frequency of infection caused by such bacteria may be low (Stenehjem and Rimland, 2013). Finally, while a bug may be resistant to a particular drug, it may be responsive to other drugs, requiring categories of ‘extremely drug resistant’ and ‘pan resistant’ infections.

The above description demonstrates the multiplicity of AMR (Mol, 2002); definitions of AMR could be as numerous as drug-bug combinations exist, articulated in clinical, microbiological and molecular registers. Nonetheless, AMR needs to be counted in order to demonstrate its relative importance (Adams, 2016). A key commitment from UN Member states has been to set up surveillance on AMR. Once AMR can be counted then it is possible to account for action upon it. This mirrors Foucault’s proposition of the role of statistics in shaping public health (Foucault, 2008). Currently, however there is limited data on AMR—in any of its possible definitions—outside of well-resourced tertiary care settings or research studies. The WHO (2017) AMR surveillance report demonstrated the paucity of data around the globe that count AMR (World Health Organization, 2017). It also demonstrated the heterogeneity in what is captured between countries under AMR. The multiplicity within the term AMR may explain why it has been difficult to define indicators to capture its prevalence in a meaningful way across species, medicines and settings. Existing surveillance systems typically collate microbiology data of isolates from hospital patients. A key challenge in counting AMR is to expand such surveillance globally, and beyond the hospital setting. At the moment, estimates of the AMR burden in LMICs is derived through a series of extrapolations—for example the proportion of neonatal sepsis deaths attributable to drug resistant infection, estimated as 214,500 in 2012, based on an expected proportion of all neonatal deaths attributable to severe infection, and of those, attributable to a drug resistant infection to the first line treatment (Laxminarayan et al., 2016). Even with planned investment in laboratory capacity for microbiology, from the UK’s Fleming Fund, the US Centres for Disease Control and others, surveillance is unlikely to expand beyond a reference site model in most low resource settings (Seale et al., 2017), leaving population levels of AMR, and clinical impact of drug resistance relatively unknown. The scale of the AMR problem is inferred rather than known, even in relatively high resource settings. In the US, around 25,000 excess deaths have been associated with AMR yearly, but even there it is accepted that ‘such estimates depend heavily on the type of included infections and resistance mechanisms, and the extrapolating techniques used’. (Pires et al., 2017). When looking at finely grained patient data, a review of 15 years of data from 2001 in Marseille, France, found that extremely drug resistant bacteria—where bacteria were resistant to more than two antibiotics—were uncommon (37 of 27,681 inpatient bacterial infections) and of these cases four cases died, three attributable to other causes (Abat et al., 2017). However, the stark contrast in incidence of infection between this setting and many LMICs leaves the question open as to transferability of these findings across settings.

Generating capacity for AMR surveillance is a clear priority. However, this will take substantial time, and resources. Predicting the course of AMR is argued as key to prepare for the potential crisis. Even with good data, however, the trajectory of resistance has been shown to be non-linear, dependent on drug-bug combination but also context. When the Marseille group reviewed hospital data over 15 years from 2001 to look at trends in AMR (Abat et al., 2017), they found that MRSA actually decreased, and there were no changes in levels of vancomycin or imipenem resistance in clinically important bacteria. With antibiotic stewardship, drug resistant infection has been found to decrease in some hospital settings (Baur et al., 2017), although the relationship is not always so clear, as susceptibility does not always rebound after drug pressure is reduced (Burke, 1998). Thus, the future direction of ‘AMR’ as a collective category is hard to predict on a granular level.

In summary, stabilising AMR through actuarial accounts has been met with definitional, technical and resource challenges. In the places where we do have figures, they are relatively small compared with other health concerns. In order to propel AMR to a global political stage, and to compete with other disease that have gained attention and funding on a large scale, AMR has required a different form of articulation.

### The sentinel approach: projecting AMR

We have seen that AMR is difficult to define in the singular; the phenomenon captures multiple processes, bugs and drugs. We have also seen that it is difficult to count, both because of its multiplicity and due to resource and technical limitations. Nonetheless, it has been possible to evoke projections of AMR—of what it might be—and therefore to stabilise it as a threat in the future tense, that must be acted upon in anticipation.

An increasing number of public, private and multilateral institutions have now published reports on AMR. A familiar structure can be observed in each, through which AMR is described as a major health threat, driven by antibiotic overuse, and a description of the consequences of AMR and set of solutions is then outlined. Quantification of the current scale or nature of the AMR problem is notably absent; reports allude to the ‘lack of data’ whilst emphasising a ‘certain threat’. The UK Government-commissioned O’Neill Review on AMR provided a projection that, if left unchecked, 10 million people would die per year from AMR by 2050 (O’Neill, 2014). While the science behind these figures has been contested (de Kraker et al., 2016), and many openly recognise the figures as of more political than scientific value, the figures have been widely used across science and policy, including as ‘underestimates’. In their follow-up book, O’Neill and colleagues provide images of the shape of the curve that AMR impact could take into the future – although this is in economic rather than morbidity or mortality terms (Hall et al., 2018). The authors echo a dominant sentiment in the field that ‘we should always try to improve predictions but we should take action now.’ This sentiment of the need for action regardless of the accuracy of the threat is common across AMR discourse, for example when arguing for more investment into AMR monitoring architecture, Wernli et al. (2017) state that ‘While the long-term impact of AMR on human societies remains uncertain, the conservation of antimicrobials’ effectiveness has become an urgent priority’ (p. 1).

The way AMR is framed as a threat, a potential catastrophe, echoes the nature of other pandemic threats in recent times. The discourse is remarkably similar in form to that of pandemic influenza (Caduff, 2015) as well as SARS, Zika (Lakoff, 2017) and Ebola (Kelly, 2018). Where AMR is described as a ‘growing spectre’ (Talkington, 2017), H1N1 was described as a ‘killer strain lurking in the shadows.’ Caduff (2015) observes that a key aspect of framing disease as a threat is the generation of a ‘public culture of danger’, through which he argues subjects of liberal rule are systematically reminded that life is under threat; and, following Foucault (2008), that the fear of such existential threat ‘is at the heart of the modern social contract and the formation of modern political communities’ (Caduff, 2015, p. 189). The resulting anticipatory affect can be understood as part of practices of making futures present—by formulating life as contingent, subject to future surprises, forms of pre-emption and pre-mediation are required as anticipatory action (Anderson, 2010; Cooper, 2006).

As well as the threat of AMR forming a biopolitical project through looming health crisis, an area that has been particularly developed as a threat in AMR compared with other pandemics is the impact on economies. Foregrounded here are the potential economic costs of AMR to health and healthcare in given future scenarios. While earlier economic forecasts found the additional costs associated with AMR compared with susceptible infections to be relatively small (Smith and Coast, 2012), more recent estimates are substantially larger, drawing on a different set of assumptions. As Bruce Braun has reflected, the shift from calculable disease risks to anticipation of potential or ‘virtual’ events brings challenges for planning; he asks ‘how does one bring the ‘unspecifiable’ future-to-come within the realm of economic and political calculation? By definition the virtual is incalculable.’ (Braun, 2007, p. 19). In AMR, a way forward was forged by British economists engaged in producing cost-impact estimates for AMR, Richard Smith and Jo Coast, who wrote in 2012 that ‘the cost of resistance needs to be high *now* to justify greater restriction on use of current drugs’ (Smith and Coast, 2012, p. 8), and further argued that estimates ‘must encompass the costs that might relate to the loss of modern healthcare’ (Smith and Coast, 2013, p. 2). Their example case of hip replacements as an example of the multiple points at which AMR could cost the health system has been adopted widely to illustrate the potential catastrophic costs of AMR (European Commission, 2016; World Health Organisation, 2016; Department for Health, 2013). The authors go further to ‘speculate’ about the impact on productivity and wider societal costs, proposing that to include these costs, and a scenario where no antibiotics work, would allow estimation of ‘the full *potential* economic costs’ (sic). They argue that ‘rather than see expenditure on antimicrobial policies as a cost, we should think of it as an insurance policy against a catastrophe; albeit one which we hope will never happen’ (ibid. p. 2). Thus, the criteria for economic costing was widened considerably, although also with considerable uncertainty. When the O’Neill report authors commissioned teams from KPMG and RAND Europe to calculate worldwide estimates of the impact of AMR, they followed the principles of this model, resulting in projections of cumulative cost to global economic output of 100 trillion USD by 2050 if no action is taken (O’Neill, 2016). Following the O’Neill Report, in 2017 the World Bank published a report on Drug Resistant Infection with the subtitle ‘A threat to our economic future.’ The report is based on economic simulation tools and provides a vision of ‘AMR’s destructive impacts on the global economy… if adequate measures aren’t taken to contain the AMR threat.’ The report’s executive summary emphasises that ‘putting resources into AMR containment now is one of the highest-yield investments countries can make’ (World Bank Group, 2017). This rhetoric echoes the policy rhetoric that ‘we cannot afford to return to a pre-antibiotic era’ (Tripartite Collaboration on AMR, 2016).

The emphasis on investment in health issues on a global scale is not new or unique to AMR. It is an emphasis that has shifted the shape of international health efforts since Rockefeller applied his business models to his Foundation, most notably in the *investment* into countries to support malaria control (Eckl, 2014). However, the application of the models used in the now dominant form of investment—hedge funds—to health is relatively recent. Thus, not only is the principle of supporting other countries’ health issues seen as an investment with return for the supporting country, but the mathematical models and assumptions that go along with investments into speculative futures are also being applied. George Osborne, UK Chancellor of the Exchequer under David Cameron’s Prime Ministership, is quoted in O’Neill’s Superbugs book as saying, ‘there is a growing recognition of the financial costs of failing to tackle antimicrobial resistance and the need for financial expertise in developing the solution.’ (Hall et al., 2018, p. 52). Encouraged by the desire for funding technological solutions to AMR venture capitalists in the AMR space are identifying ‘opportunities’ for investment. One major opportunity is for diagnostic testing, with a potentially global market for tests for surveillance and treatment. Thus, the ‘sentinel’ approach to disease surveillance emerges where we invest in vigilance and speculative future events (Anderson, 2010), fluently incorporating both the language and the capital of the current financial sector’s models. Emerging from these models are forecasts that can be hedged upon, and with the emphasis on technological development as a solution to AMR, private investors are able to anticipate different potential futures upon which they may place options.

The language of a ‘sentinel’ approach also reminds us of the securitised nature of the AMR phenomenon. The projections of and preparedness for threats stand to protect publics from danger (Caduff, 2015). This danger in AMR is often personified in the form of ‘superbugs’ with which we are to wage war (Nerlich, 2009). An increasingly popular approach, also borrowed from war, when preparing for health crises and disaster management is simulation (Lakoff, 2007). Deployed in the 1980s for Ebola ‘war games’ (Garrett, 1994), simulations have since been used as modes of preparing for potential pandemics such as smallpox, anthrax and avian influenza (Lakoff, 2007), and are common in preparing for zoonotic disease outbreaks both in high income settings (Keck, 2018) and in national programmes for example in Uganda’s ‘One Health Strategic Plan’ (Republic of Uganda, 2018). Lakoff (2007) has drawn attention to the way priorities and resources are re-designated as a result of simulations, ‘by making infrastructural vulnerabilities visible’ (p. 266). Frédéric Keck (2018) has argued that, as well as operating as pastoral techniques of power, we can also understand the work done through these simulations as ‘cynegetic’, building in uncertainty at the borders between species (p. 15). As simulation exercises begin on the topic of AMR, for example at the G20 meeting in Argentina in October 2018 (see, e.g., Department for Health and Social Care, 2018), these efforts seem at this point to orientate simulation more as a discussion and galvanising tool, to re-emphasise threats, which at once opens up the uncertainty and potential catastrophic scale of AMR, and at the same time creates certainty in reinforcing messages that action is required now.

Finally, it can already be observed that AMR is understood as a threat not only to health, to economies, to security, but also to modernity itself. Statements that AMR could hail the end of modern medicine echo through the media, and are common in speeches at both policy and research events. For example, at the UK House of Commons Enquiry into AMR, the Chief Medical Officer warned that ‘We will lose modern medicine’, the ability to undertake major surgery, cancer treatment and transplants, ‘There will be a lot of suffering and modern medicine will be lost’ (House of Commons, 2018, p. 3). At stake are many of the ways we care for our bodies, ways that we have invested in and developed infrastructure to support in the modern era. The potential loss of antimicrobial efficacy also represents a less-vocalised threat to modernity beyond health care—to our modes of production which we become increasingly aware are reliant on antimicrobials. Thus the fight *against* AMR can be read as much as a fight *for* modernity.

Therefore, when AMR is presented as one of the most pressing global threats—articulated in terms of a threat to health, economies, security and modernity—we see bound together a classic biopolitical phenomenon in a neoliberal framework. So that despite the ambiguity of the AMR concept, and our lack of ‘actuarial’ knowledge of its current scale or future impact, an imperative to act on AMR has become clear. Just as we saw with the spectre of avian influenza (Davis, 2005), the communities of practice emerging around the policy-science-industry nexus relating to AMR, and the consequent multilateral statements and commitments, are testament to the effective construction of AMR as an object that it is agreed must be acted upon.

## A problem of connectedness; individualised solutions

‘People need to be brought on-board, so we are together in the one health approach’ I hear from an East African civil servant, ‘but implementation is a challenge with irrational use of drugs by villagers’. The rhetoric of a One Health approach, accompanied by frustration at human behaviour, echoes many conversations and consultations I’ve engaged in with experts and policy makers about the intractability of the AMR problem. What is the relationship between these two conceptualisations?

*Fieldnotes* *East Africa 18, 2018*

In the previous section we have seen that AMR has been stabilised as an object in the form of a threat, and that this has been important for galvanising support—‘Why we need to act now: We cannot afford to return to a pre-antibiotic era’ (Tripartite Collaboration on AMR, 2016). We have also seen the concern that political commitment should be translated into implementation—‘only 5% of countries have a multisectoral AMR action plan that has been implemented with identified funding sources and monitoring processes in place’ (Wellcome Trust, 2016a). In this section, I explore two frameworks for action that are prominent in global discourse. The first is the narrative that explains AMR as a One Health problem, building a growing number of sectors into the definition of the problem, and therefore as points to locate solutions. The second is recourse to established rhetoric and instruments of behaviour change for formulating solutions. While the first strengthens new paths of connection between sectors, between humans and ‘nature’, and between the local and the global, I argue that the second obscures this understanding of connectedness by foregrounding individual responsibility for the AMR phenomenon.

### One health: AMR as a problem of connection

**Fig. 1:**
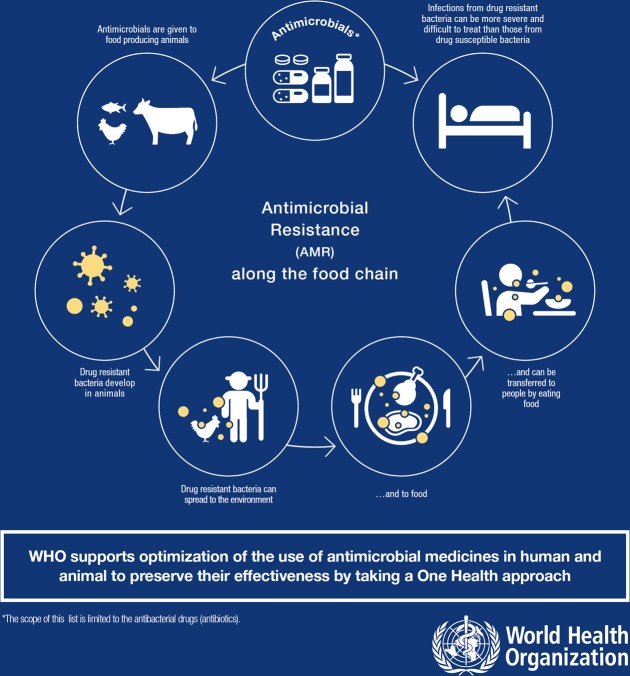
Antimicrobial Resistance in the Food Chain infographic from WHO. This figure is covered by the Creative Commons Attribution 4.0 International License. Reproduced with permission of World Health Organisation from “Infographics: Antibiotics in the Food Chain. WHO list of critically important antimicrobials (WHO CIA list)—5th revision” https://www.who.int/foodsafety/areas_work/antimicrobial-resistance/AMR-food-chain-infographics/en/ Copyright © WHO (2017), all rights reserved

Today, addressing AMR is often described as requiring a ‘One Health’ approach—typically defined as ‘multisectoral’ (World Health Organisation, 2015). This means understanding drivers of AMR as located within and between agricultural, human health and environmental domains. Diagrams illustrate the emergence and circulation of drug resistant bacteria through livestock, food, water, hospitals, people, sewerage and so on, as shown in Fig. 1 (see also, e.g., Coutinho et al., 2013; Walsh, 2018) as well as showing the flow of antimicrobial drugs through these same channels (see, e.g., BioMerieux figure ‘The Spread of Antibiotic Resistance’ (accessed October 2018)). These illustrations emphasise the many points of connection within particular ecosystems that can be understood to increase risks of AMR.

The One Health approach to AMR also promotes interdisciplinary collaboration (European Commission, 2017; UK Government, 2013). Developments in the science of AMR allow us to understand how resistance travels between microbes, and we have become increasing attuned to microbial ecologies through concepts such as the microbiome, evoking a heightened sense of interconnectedness between humans and the natural world (Landecker, 2016). Therefore, whilst rhetoric that utilises metaphors of war implies an ‘us’ and ‘them’ in fighting resistant bacteria, the One Health framing draws together an imagery that begins to depart from classic formulations of infectious disease control. For much of the enlightened scientific era, our imagery of infectious disease has relied on compartmentalism of human, animal, insect and environmental realms. Characterised by linear arrows and cyclical feedback loops, infection of humans is depicted in terms of pathogens traversing these realms. In this perspective, humans are considered separate (and separable) from the ‘natural’ reservoirs of infectious disease (Lynteris, 2017). Through apparatus such as DDT, extermination, physical barriers, characteristic of ‘hygienic modernity’ (Rogaski, 2004), we were able to visualise a disease-free humanity separated from nature (Lynteris, 2016). Research in a One Health framework does begin to question these imagined boundaries, with new ways of understanding infection, such as ‘folded’ life (Hinchliffe and Ward, 2014). As people struggle to capture the intricacies of AMR with a One Health approach, diagrams become ever more complex, and it becomes clear that depictions of arrows, drivers, factors, as linear processes of infection from the outside in, become increasingly insufficient to account for AMR in the context of these dissolved boundaries.

A One Health approach to AMR often also incorporates ‘One World’, reflecting the global nature of the problem with connections through food systems and human travel (Robinson et al., 2016). We often hear and read that ‘AMR does not respect borders’ (Wellcome Trust, 2016a; World Health Organisation, 2018a). In an article posted on 10 October 2017, the Pew Charitable Trusts released an animated map showing the global spread of resistance gene ‘New Delhi metallobeta-lactamase 1’ or ‘NDM-1’ (Talkington, 2017). Tracking the publications of findings of this gene from 2006 to 2015, the animation colours in the greyed-out map as years progress—first India becomes red, then a series of other countries each year, until in 2015 there are 80 red countries. The accompanying commentary remarks, ‘These outbreaks are a sobering reminder that when antibiotic resistance develops anywhere, it is a threat to people everywhere.’ (ibid). Another recent example raising concerns of the spread of an emergent resistant gene is ‘mobilised colistin resistance 1’ or ‘MCR-1’, identified in pigs and humans in China in 2011 and subsequently identified in countries across five continents (Wang et al., 2018).

While the One Health approach to AMR mainly focuses on drawing attention to interconnectedness between domains—whether sectoral, disciplinary or countries—it also has allowed for analyses of reasons for the emergence of AMR, drawing attention to the ways we connect with each other and with nature. The relationships between antimicrobial use and resistance remain mysterious, with analyses suggesting a weak link for some drug-bug combinations at both a macro and micro level (Collignon et al., 2018; Caudell et al., 2018). Nonetheless, there is an increasing understanding of AMR as an anthropogenic problem, a consequence of our collective reliance on antibiotics (Lee and Motzkau, 2013). Such a perspective is palpable in the mainstream, following the path paved by climate change (ibid). For example in their call for a global governance framework, participants from a Leeds Castle meeting in 2018 led by Dame Sally Davies note that, ‘The emergence of resistance is a natural phenomenon but is accelerated by a complex combination of human activity in health care, agriculture (including animal husbandry, aquaculture, and crops), and environmental contamination’ (Rochford et al., 2018, p. 1977). Thus, we begin to see sensitivity to material-biotic interdependencies emerging in global level discourse, and we see the framing of One Health being mobilised to encompass numerous registers of connectedness in understanding AMR. To a degree, this suggests a move towards understanding health as no longer bounded and separable from other arenas of economics and politics, and humans as no longer separable from nature. This can also be understood in relation to the ‘sentinel’ model described above, in which bodies are imagined as at risk of surprise pathogenic attacks in a chaotic and unpredictable molecular world, as Braun observes, ‘a body understood in terms of a general economy of exchange and circulation, haunted by the spectre of newly emerging or still unspecifiable risks’ (2007, p. 15).

### Behaviour change: individualised solutions

Given this rhetoric of AMR as a One Health issue, advocating for recognition of complexity and connectedness that requires a multi-sectoral response, it is curious then to observe that the behaviour change of individuals is so frequently cited as a key solution to AMR. For example, Public Health England’s *Keep Antibiotics Working* campaign specifically ‘focuses on the personal risks of antibiotic resistance’ in an attempt to reduce demand for antibiotic prescriptions from doctors (Public Health England, 2017). Behavioural targets are primarily reduction of antibiotic use by patients, prescribers and farmers to reduce drug pressure on microbial populations, and handwashing and allied hygiene behaviours to prevent the spread of AMR. The upsurge in interest in AMR has revitalised the rational prescribing movement, energised by the behavioural economics models (Rynkiewich, 2018) that are increasingly influential in social policy in the UK and US to ‘nudge’ people to ‘do the right thing’ (Matjasko et al., 2016). There is now increasing interest in WASH (water, sanitation and hygiene) and ‘biosecurity’ behaviours in the human and animal health domains, respectively. For both antimicrobial use and hygiene interventions, there is increasing recognition that existing evidence base is unable to provide confidence in guiding effective and cost-efficient action. Several reviews have now underlined the ‘mixed results’ of evaluations that are unable to identify a desired recipe for successful behavioural interventions on antimicrobial use (Price et al., 2018; Davey et al., 2017; Arnold and Straus, 2005; Van Dijck et al., 2018). Nonetheless, the precautionary principle, a concept that relates to the sentinel approach explored above, requires action now, while further research is commissioned to hone in on the best ways to change behaviour.

**Fig. 2:**
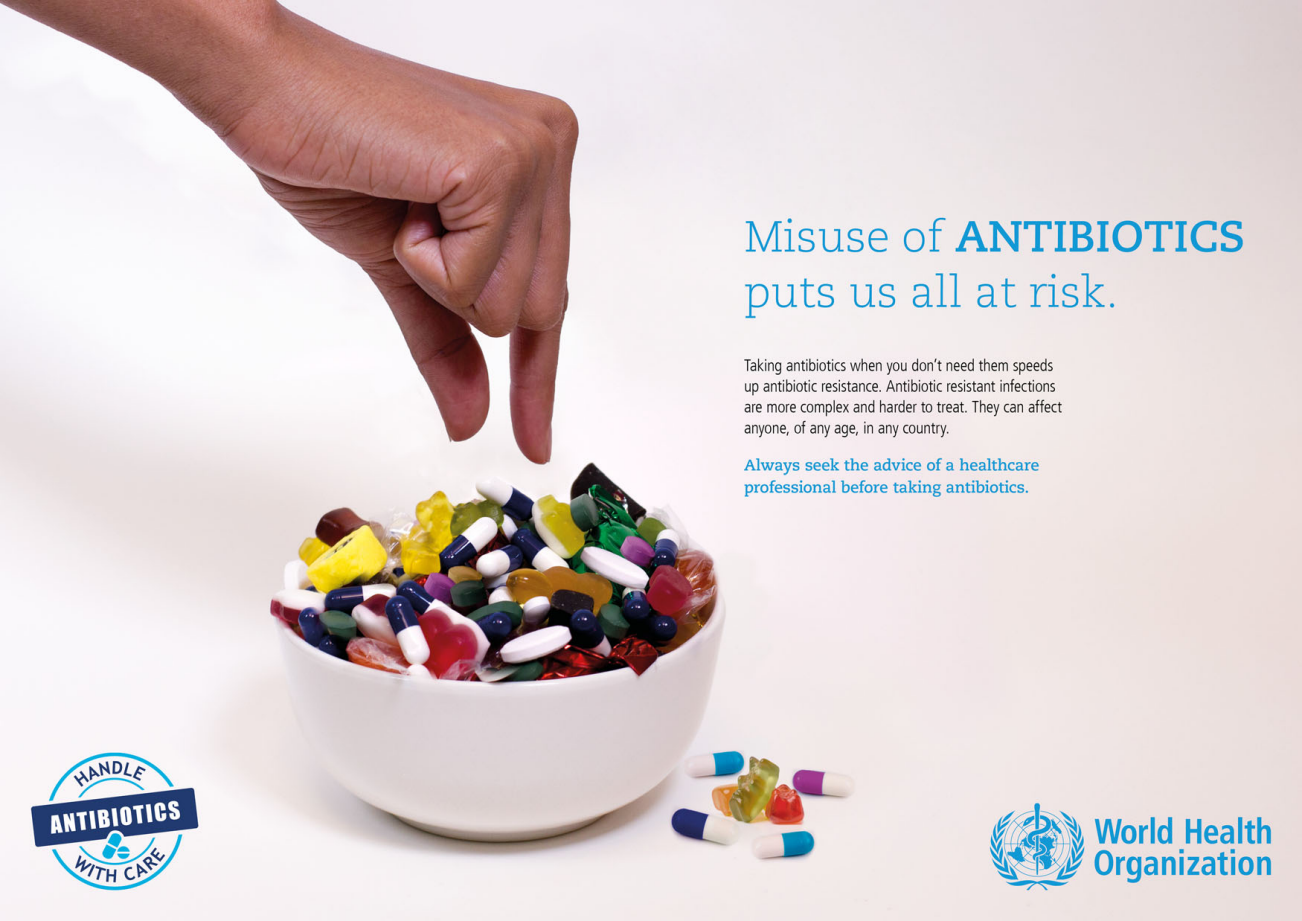
WHO Poster for World Antibiotic Awareness Week (2017). This figure is covered by the Creative Commons Attribution 4.0 International License. Reproduced with permission of World Health Organisation from ‘World Antibiotic Awareness Week 13–19 November 2017' https://www.who.int/campaigns/world-antibiotic-awareness-week/2017/posters/en/ Copyright © WHO (2017), all rights reserved

Current interventions for reducing antimicrobial use revolve around raising awareness and what is now widely referred to as ‘stewardship’. Campaigns and programmes orientate around individuals realising their responsibility to prevent a future tragedy that may occur due to their unnecessary use of antimicrobials today. Thus, materials are often designed to communicate risk, linking the future to actions today. For example, ‘Misuse of Antibiotics puts us all at risk’ (Fig. 2, WHO, 2017). And materials communicate the choice that an individual has—e.g., the choice of an individual to take a pill like they might take a sweet (Fig. 2). The premise of such communication includes not only that the audience is ignorant of the topic, but also that the reasons for their behaviour emanates from a position of choice. They could choose not to, as easily as choosing not to eat a sweet. Not only does this fall into the common trap of isolating behaviour from context (the reasons for taking or prescribing an antibiotics are invariably dependent upon non-behavioural factors, and are often beyond the control of individual patients or prescribers—(see, e.g., Haenssgen et al., 2018; Pearson and Chandler, 2019)) but it also imagines that taking antibiotic medicines is a decision that is taken as unthinkingly as eating confectionary.

Underlying these behaviour change models—both in the antimicrobial stewardship and hygiene domains—is the assumption that action is instigated by cognitive or habitual functions of individuals. Moreover, it is often assumed that existing practices are rooted in erroneous beliefs or problematic culture that require intervention (Haddad, 2016). The focus on individuals as targets of interventions for change can be understood as part of a liberal framework that tends to locate risk and responsibility at the level of citizens (Petryna et al., 2006). Following in the footsteps of ‘lifestyle choices’ as a framing that recast responsibility towards individuals and away from the state (Lupton, 1995), at home in the rhetoric of choice (Mol, 2008) and self-improvement (Türken et al., 2016), and resonant with the logics of global health metrics and accountability (Adams, 2016) the thrust towards ‘behaviour’ in AMR interventions allows for deviant individuals to come into view as amenable to evaluable intervention while expectations from the state are obscured as unfeasible and idealistic. Political and infrastructural issues, that the One Health framework would indicate are likely to underlie AMR risk, are cast as ‘complex’ and ‘not amenable to change’, to be circumvented with simple technologies as repeatedly observed in global health (Street, 2012; Beisel et al., 2016).

Thus, while a review on country AMR progress by the WHO (2018b) notes ‘The necessary changes to global antimicrobial use exceed what can be achieved using individually targeted behaviour change strategies’ (p. 3), the report goes on to emphasise the need for progress ‘as most countries have not yet launched nationwide, government supported campaigns on AMR awareness in human health, nor have they implemented strategies to change behaviour regarding AMR in target groups in human health’ (p. 12). Taking together the uncertainty in the evidence base of behaviour-oriented interventions, and the evidence used to build the One Health argument that suggests AMR as a systemic issue of connectedness, rather than individuals, one might expect an increase in activity and research to address ‘structural’ level issues that shape possibilities for action at a local level. This is occurring to a certain degree, with an ongoing review of governance interventions (Rogers Van Katwyk et al., 2017) and for example through the Wellcome Trust’s (2016b) proposal for ‘enhanced ‘gating’ of antibiotics, ‘so more use is routed through healthcare professionals and over-the-counter use is minimised’ (p. 2). Although such proposals are challenging to negotiate given parallel concerns around access to lifesaving antimicrobial drugs (Laxminarayan et al., 2016), such concerns might equally be applied to individual-oriented interventions (see, e.g., the unintended consequences of malaria rapid diagnostic tests on access to antimalarials (Hutchinson et al., 2017a, 2017b)). Nonetheless, analysis of current discourse suggests most often a doubling-down on the individual; following the pastoral model of biopolitics characterised by Rose (2001) and which stands in contrast to the securitised form of geopolitics described in the One Health framework (Braun, 2007). Adding to the ongoing focus on cognitive interventions, there is increasing interest in targeting affect or emotion for behaviour change. For example, in a review on hand hygiene interventions, Wilson et al. (2011) propose that ‘interventions that provoke emotive sensations (e.g., discomfort, disgust) or use social marketing may be the most effective’ (p. 119). Similarly, a review on the nature of mass media campaigns on AMR concludes that ‘further research is needed to systematically illuminate and capitalise upon the use of affect to effect behaviour change concerning antimicrobial stewardship.’ (Langdridge et al., 2019).

## Antibiotics as Infrastructure

There is a recipe for the rapid growth of pig and chicken farming in Uganda: smaller spaces, larger imported breeds, intensive cleaning, imported feed concentrates, and antibiotics. ‘I use a lot of medicines, including antibiotics', explains a middle-aged gentleman who like many others is new to farming, having left employment in the civil service. ‘If I do not, then I cannot make a profit'. He and his fellow farmers describe how the recent push to commercial rather than subsistence farming reflects a number of societal shifts. There is liberalisation, which they say means that they as members of the population now shoulder greater risks—and potential rewards—with commercialised farming models. Then there is modernisation, which they explain increases demand for eggs, chicken and pork, ‘so there is a push to enhance how much your chicken can produce'. Then there is standardisation, whereby a particular sized chicken is given a set price on the market, and if you can produce it in 4 rather than 6 weeks then you can get an edge on balancing your books. In each case, we learn that antibiotics play an important but often invisible role.

*Fieldnotes* *East Africa 152, 2018*

German scientist and Nobel laureate Paul Ehrlich used the term *Zauberkugel* (Ehrlich, 1913), or ‘magic bullets’, to refer to substances that could kill disease-causing microbes without harming the host. The term captures the imagination as an ideal targeted intervention, drawing attention to the effectiveness rather than the effects of these magical substances. Unwanted effects blur out of focus. They are ‘side’ effects, mundane and uninteresting in contrast with the magic of high efficacy. Whilst retaining their mystique and desire as powerful substances, the mass production of antimicrobials and possibilities they presented rapidly moved them into the fabric of lives and healthcare systems around the world (Podolsky, 2015). Antimicrobials were soon seen as essential to human life, with access to these medicines enshrined as primary health care in the declaration signed by 134 countries from around the world at Alma Ata in 1978 (World Health Organisation and United Nations Children Fund, 1978). Along with the push to ensure access to these medicines, came a realisation that recipients may not wish to use the drugs, or may use them in ways other than those defined within biomedical practice. The push for the rational use of medicines therefore emerged in parallel with the drive to increase access (Podolsky, 2015). However, as described above, the concept of rational medicines use has focused on behaviour of individuals—patients, prescribers, farmers—and has not attended to interconnections with wider changes in social, political and economic structures and relations. These relations into which antibiotics have become intertwined have largely been considered the domain of other analyses, disciplines, policies—disconnected from the materialities of antibiotics. However, in her clarion call to attend to AMR, or witness ‘the end to modern medicine’, Dame Sally Davies draws our attention to the threat AMR poses to the whole apparatus of modern medicine. And one might extend this argument to see these concerns as a response to a threat to modernity itself.

The present moment of antimicrobial resistance as a major topic of global concern can be understood to represent a moment of inversion—when antimicrobials have been rendered visible where previously they have been a part of the woodwork. In their work on classification systems as infrastructure, Bowker and Star (2000) observe that good usable systems ‘disappear almost by definition. The easier they are to use, the harder they are to see’ (p. 33). Such infrastructure comprises materials, information, ordering. For example, the availability and usability of research instruments and subjects shapes what science is constructed; the supply chain, techniques and subject handling methods are invented alongside biology’s conceptual frame. They argue that to render such infrastructure visible ‘means learning to look closely at technologies and arrangements that, by design and by habit, tend to fade into the woodwork’ (p. 34). The idea of infrastructure defining what is possible follows Becker (1982) who observes how infrastructure is deployed as ‘conventions and constraints’ such that the length of musical concerts and sizes of paintings are set within particular parameters. Inversion of the status quo is one way that the woodwork may be rendered visible. An 8-foot painting or 8-h musical would render visible the roles of parking attendants, ticket takers, theatre rentals, the positioning of paintings on walls, the size of rolls of canvas, the skills of framers, the very doorways of museums and galleries. Thus, the practice of inversion brings to the fore the arrangements of objects, people and processes that may otherwise go unobserved and yet shape possibilities for the ways things can be done and conceived. The present awareness of antimicrobial resistance appears to have produced an inversion whereby antimicrobials, and their attendant relations and processes, have come to the fore. This enables analyses of the possibilities, conventions and constraints that have hitherto been taken for granted as common sense, and the potential for reshaping these into the future.

There are numerous ways in which one might productively explore this inversion of infrastructure presented by AMR. Here I propose that analyses of antimicrobials as material, affective and political infrastructures is valuable to an effort of rendering visible the work of these medicines.

### Material, affective and political infrastructures

What does the threat of antimicrobial resistance draw our attention to in the material infrastructural arrangements of domestic, health and industrial settings? Historical work has highlighted the role of hygiene and sanitation infrastructure in bringing about infection control (Illich, 1976), on the tail of which antibiotics are also understood to have enabled reductions in infectious diseases (Headrick, 1981). To what extent antibiotics *replaced* or *reshaped* the material infrastructure in maintaining disease control has been less directly studied. Robert Bud draws our attention to the dramatic increase in the number of beds on British medical wards with the founding of the National Health Service in the mid twentieth Century, when ‘the threats of cross-infection created by this increased turnover were again managed by antibiotics’ (Bud, 2006, p. 197). The roles antibiotics now play in disease prevention for surgery and chemotherapy, and the attendant concern about the impact of drug resistant infection on the ability to carry out these procedures and processes in the speedy and relatively uncontained way they currently occur (Davies, 2013) suggests that antibiotics have become part of the health infrastructure such that they shape possibilities and constraints in pathways to health. With rising concerns of antimicrobial resistance in humans, relatively greater attention has been paid to the ways that antibiotics have been deployed to enable industrialisation of agriculture, particularly in livestock. Nonetheless, such investigation is often cut short by the ‘multi-sectoral challenge’ of the requirement for national productivity often contingent upon agricultural production. Antibiotics are therefore switched-in for biosecurity measures that attempt to prevent and contain infection whilst maintaining intensive farming. Such measures build on the same tenets of standardisation of material as antibiotics allow, rather than folding in diversity (Hinchliffe and Ward, 2014). Attention to antibiotics as material infrastructure, and the inversion posed by AMR if these substances were to be removed, prompts further questioning of how materials are currently arranged and entangled with antibiotics.

Bringing to the fore antibiotics as affective infrastructure allows for analyses of the ways in which these substances enable and define relations—between people, organisations, countries and so on. One of the most obvious relations that AMR has brought to the fore is that of patients and prescribers—allowing us to see how these medicines have themselves become a form of care, such that in the absence of one medicine, providers often feel compelled to prescribe another (Chandler et al., 2017; Hopkins et al., 2017). Antibiotics have also shaped relations to biomedical institutions—such that with AMR, hospitals may be transformed from being seen as lighthouses of therapeutic modernity to hothouses of infection (Gradmann, 2017). They also allow us to understand what is at stake in the absence of an antimicrobial prescription—which renders visible relations with work places, expectations of productivity and the ways that antimicrobial prescriptions have been legitimising illness and providing space for recovery. An exercise to explore this further might be to follow the impact of prescribing ‘rest’ in lieu of an antimicrobial—and to thereby explicate the layers of social and economic systems that make returning to work an imperative. Such an inversion has been recently proposed by physicians observing a problem of *presenteeism* amongst health care workers which propogates disease in hospital environments, caused by sick leave policies and the value placed on ‘selfless-acts’ of continuing to work whilst ill (Chow and Mermel, 2018). And whilst antimicrobials may allow for humans to become shaped into more reliable and productive units of labour, they can also be understood to enable animals to become more reliably productive, as well as to become more standardised units of production. Thus, it is instructive to explore the pharmaceuticalisation of labour through antibiotics. In a similar vein, the economic concerns about the impact of AMR on the workforce indicate a concern over the potential slowing down of life. We become aware that antibiotics enable the current tempo of life—indexed to productivity and consumption, and entwined as much with moral as financial economies. Standardisation and scale also become a part of this story, as we trace how antibiotics have become part of the apparatus of global standards—as interfacing objects that enable exchange of things or ideas to occur—we become able to envision the wider imperatives at play in the systems into which antibiotics have become a lynchpin. Empirical research that seeks out the ways in which antibiotics are entangled with these values of tempo, production, standardisation and scale will be valuable in explicating the ways these substances form relational infrastructure across the globe today.

The exploration of the roles of antibiotics as affective infrastructure also opens-up for analysis the ways they make possible particular political-economic values in the context and drivers of urbanisation and globalisation. They enable, for example, the revisiting of medicalisation—the encroachment of biomedicine into areas hitherto conceived as outside of the medical domain. The process of medicalisation can be understood to have been shaped by values and social policy established in the Enlightenment period of 17th–18th Century, principally ‘faith in the progress and perfectibility of society with the help of science and technology’ (Risse, 1992, p. 150). For Enlightenment medicine, a key objective was ‘the displacement of such pessimistic concepts of sickness for more hopeful outlooks enshrined within new biomedical models of health and disease. Fatalism and ignorance in health-related matters had to be overcome’ (ibid, p. 154). While at this time, the focal point of medicalisation was the professional expert who could deal with health-related problems, and more recently pharmaceuticals have been observed to take their place (Samsky, 2015), the framing of hope of a disease-free humanity remains integral to biomedicine today. In their history of acute bronchitis (cough) management in Britain through the twentieth Century, Macfarlane and Worboys (2007) illustrate how antibiotics enabled particular values in human productivity to develop, allowed for a more medicines-as-prevention strand to develop within healthcare, as well as how antibiotics became entangled with the ways the pharmaceutical industry could engage with a nationalising health system. Thus one can unpack the roles of antibiotics in co-producing our modern political economies, but also one can see how these political economic values have become part of how we understand and talk about antibiotics, and resistance, mobilised in metaphors of war (Nerlich, 2009), interchangeably with migration (Brown and Nettleton, 2017), capitalism (Brown and Nettleton, 2018) and entangled in the project of modernism itself (Hutchison et al., 2018). Thus, following Dewey (1927) and Collier et al. (2017), understanding antibiotics as infrastructure in these ways enables us to ask what sorts of publics, collectives, social forms and systems are brought into being by these substances?

## Conclusion

In this paper I have drawn attention to the framing of AMR discourse and its consequences. I have argued that AMR has been less easy to advocate for with an ‘actuarial’ approach and instead support has been galvanised in its formulation as a threat, through a ‘sentinel’ approach (following Lakoff, 2015). I have drawn attention to the disconnect between the configuration of AMR as a problem of connectedness through a One Health framework, and approaches to implementation that most often target individual behavioural change. These observations allow us to explore what happens if we use different models to envision AMR which are less speculative and more actuarial? And what happens if we take seriously ‘one-health’ as a mode of governance as well as a way of understanding the problem, or if we seek to dis-embed antibiotic dependence from within neoliberal society? I propose that to explore antibiotics as infrastructure resists the micro/macro division implied in existing discourse, rather operating in a mode of entanglement. AMR presents an inversion of the current status quo of biomedicine and beyond, rendering visible in this moment the ways in which our lives are contingent upon antimicrobial medicines: to define and deliver health care; to enable productivity of work forces, industrialisation of food other commodities; as well as making possible particular social and political values in the context of modernisation, urbanisation and globalisation. In this sense, antimicrobials can be considered as infrastructure—as usable systems that disappear unless deliberately explicated. This opens-up possibilities for reconfiguring AMR research and action by shifting the focus of attention across scales and enabling different forms of care, and different publics, to come into view. Such shifts enable us to conceive of AMR not only as ‘The End of Modern Medicine’ but as an invitation to an era of medicine beyond that defined through modernity.

## Data Availability

The original data generated during and/or analysed during the current study are not publicly available as individual privacy could be compromised but are available from the corresponding author on reasonable request.
